# Fish Swim Bladder‐Derived ECM Hydrogels Effectively Treat Myocardial Ischemic Injury through Immunomodulation and Angiogenesis

**DOI:** 10.1002/advs.202500036

**Published:** 2025-04-09

**Authors:** Yulong Fu, Canran Gao, Hailing Zhang, Jing Liu, Boxuan Li, Wei Chen, Xiuping Chen, Xue Lin, Ligang Fang, Zhihong Wang

**Affiliations:** ^1^ Institute of Transplant Medicine School of Medicine Nankai University Tianjin 300071 China; ^2^ Institute of Biomedical Engineering Chinese Academy of Medical Sciences and Peking Union Medical College Tianjin 300192 China; ^3^ Peking Union Medical College Hospital Chinese Academy of Medical Sciences and Peking Union Medical College (CAMS&PUMC) Beijing 100005 China; ^4^ State Key Laboratory of Quality Research in Chinese Medicine Institute of Chinese Medical Sciences University of Macau Macao 999078 China

**Keywords:** extracellular matrix, fish swim bladder, heart failure, injectable hydrogel

## Abstract

Injectable hydrogel implants represent a promising therapeutic approach for ischemic heart failure; but their efficacy is often limited by low bioactivity, poor durability, and inadequate injection techniques. Herein, a unique hydrogel incorporating extracellular matrix from fish swim bladder (FSB‐ECM), which has distinct advantages over mammalian derived ECM, such as low antigenicity, bioactivity, and source safety, is developed. It consists of collagen, glycoproteins, and proteoglycans, including 13 proteins common in the myocardial matrix and three specific proteins: HSPG, Col12a1, and vWF. This hydrogel enhances cardiac cell adhesion and stretching while promoting angiogenesis and M2 macrophage polarization. In addition, its storage modulus (*G*′) increases over time, reaching about 1000 Pa after 5 min, which facilitates transcatheter delivery and in situ gelling. Furthermore, this hydrogel provides sustained support for cardiac contractions, exhibiting superior longevity. In a rat model of ischemic heart failure, the ejection fraction significantly improves with FSB‐ECM treatment, accompanied by increased angiogenesis, reduced inflammation, and decreased infarct size. Finally, RNA sequencing combined with in vitro assays identifies ANGPTL4 as a key protein involved in mediating the effects of FSB‐ECM treatment. Overall, this new injectable hydrogel based on FSB‐ECM is suitable for transcatheter delivery and possesses remarkable reparative capabilities for treating heart failure.

## Graphical abstract

A unique hydrogel based on extracellular matrix from fish swim bladder, which is suitable for transcatheter delivery and possesses remarkable reparative capabilities for treating heart failure, is developed. It emerges as a multi‐effect factor profoundly influencing inflammation regulation, angiogenesis, and myocardial metabolism.

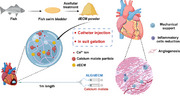

## 1 Introduction

According to WHO, there are about 56 million people worldwide suffering from heart failure (HF) in 2019,^[^ ^1^ ^]^ and this number has been increasing in recent years, leading to significant economic and societal burdens.^[^ ^2^ ^]^ Ischemic heart failure (IHF) accounts for ≈70% of the HF population^[^ ^3^, ^4^ ^]^ and is associated with a very low five‐year survival rate.^[^ ^5^ ^]^ IHF commonly occurs after acute myocardial infarction (AMI), where the coronary artery is blocked by thrombi, leading to decreased blood flow to the apex zone and resulting in cardiomyocyte (CM) apoptosis. Although stent intervention can restore blood flow, it cannot reverse the tissue injury. Owing to limited regenerative capacity, the injured myocardium is usually replaced by proliferative fibroblasts, leading to decreased diastolic and systolic function. The final treatment options for IHF include heart transplantation and artificial heart assistance, both of which are effective, but face limitations such as donor scarcity and high costs that have hindered their widespread adoption in clinical practice.^[^ ^4^ ^]^

Injectable hydrogel implants, such as alginate (ALG) hydrogels, have emerged as a promising treatment for MI since their initial demonstration in 2000.^[^ ^6^ ^]^ Extensive studies have been conducted, from basic research to clinical translation.^[^ ^7^ ^]^ A significant milestone was achieved in 2012, when the FDA approved two clinical trials utilizing Alg for MI treatment: Algisyl (NCT01311791, Lonstar Heart, Inc., USA) and IK‐5001 (NCT00847964, Bellerophon Therapeutics, USA).^[^ ^8^ ^]^ The Algisyl trial revealed an increase in the average peak VO_2_ level and a 6‐min walk test at 6 and12 months postsurgery. However, the safety risk was slightly higher than that of the control group.^[^ ^9^ ^]^ Conversely, IK‐5001, also called bioabsorbable cardiac matrix (BCM), showed no significant improvement in functional parameters, such as peak VO_2_ and 6‐min walk test, but it exhibited a favorable safety profile with a lower incidence of death and MI, albeit with a higher rate of stent re‐thrombosis, revascularization, and other therapeutic interventions.^[^ ^10^ ^]^ The differences in efficacy and safety profiles can be attributed to the Alg component and delivery method used. Specifically, Algisyl is delivered directly into the myocardium during open chest surgery and has stiffer mechanical properties than BCM which is administered via transcatheter through the coronary artery. These insights highlight the importance of both alginate hydrogel selection and delivery system adaptability for successful treatment outcomes.

To increase the therapeutic efficacy of hydrogels, numerous efforts have been undertaken, including embedding stem cells or bioactive molecules within the hydrogels prior to injection. This approach aims to mitigate ROS levels, alleviate acute inflammation, and foster revascularization after myocardial infarction (MI).^[^ ^11^, ^12^, ^13^, ^14^, ^15^ ^]^ However, despite their effectiveness, the clinical translation of these technologies remains a challenge owing to the complexity of regulatory pathways, limited off‐the‐shelf availability, and high costs. In addition, the integration of alginate with natural materials, such as dECM from porcine heart, has led to improved biocompatibility.^[^ ^16^ ^]^ However, the ECM from mammalian tissue faces several challenges, including the risk of acute immune rejection and cross‐species pathogen transmission from mammals. The fish swim bladder is an organ that aids fish in floating in water and composed of collagen, glycosaminoglycans (GAG), and elastin, closely resembled the native myocardium in terms of composition and microstructure, demonstrating excellent biocompatibility.^[^ ^17^ ^]^ Compared to mammalian tissues, dECM from fish swim bladders has several advantages, particularly in terms of low antigenicity, bioactivity, and source safety. Mammalian tissues, such as those from porcine hearts, predominantly express the α‐galactosyl epitope (α‐Gal), which is a primary xeno‐antigen responsible for acute immune rejection in humans. In contrast, fish tissues, including swim bladders, lack α‐Gal owing to evolutionary differences. This absence significantly mitigates the risk of immune rejection during xenotransplantation. In addition, as lower vertebrates, fish pose a reduced risk of cross‐species pathogen transmission compared with mammals. This characteristic further establishes them as a safe source of biomedical materials. Moreover, fish possess a remarkable capacity for cardiac regeneration compared with mammals.^[^ ^18^ ^]^ Specific proteins and amino acids have been identified in fish swim bladders.^[^ ^19^ ^]^ As reported, the dECM from fish swim bladders has been found to be rich in unique amino acids such as cystine, which is relatively rare in mammalian tissues. Cystine plays a crucial role by stabilizing protein structures through disulfide bonds and participating in redox reactions; this enhances both the stability and biocompatibility of the material. A cardiac adaptive conductive hydrogel patch based on FSB has also been developed, providing a mechanical‐electrical anisotropic microenvironment conducive to heart repair.^[^ ^20^ ^]^ Overall, these results suggest that dECM from fish swim bladders is promising for treating heart failure.

In addition, an efficient delivery system for specific injectable hydrogels is necessary to ensure both safety and efficacy of therapeutic outcomes.^[^ ^21^ ^]^ Direct injection via cardiac access is the primary method used during open‐chest surgeries. As minimally invasive techniques evolved, three alternative strategies emerged: cardiac injection through the pericardium, endocardial injection, and coronary artery injection.^[^ ^22^ ^]^ Intramyocardial injection offers strong directionality and excellent repair effects, as exemplified by Algisyl and Ventrigel; however, it is highly invasive. Transcatheter coronary injection ensures high safety; however, the retention of hydrogels, such as IK5001, cannot be guaranteed. Transcatheter intramyocardial injection, which balances safety and effectiveness, is the ideal approach;^[^ ^23^, ^24^ ^]^ however, it imposes stringent rheological requirements on hydrogels, including viscosity, rheological properties, gelation time, and stability under compression. Specifically, the hydrogel must remain in a liquid state while passing through the catheter, necessitating a suitable gelation time to prevent clotting within the catheter, yet allowing for gelation once injected into the injured tissue.

**Figure 1:**
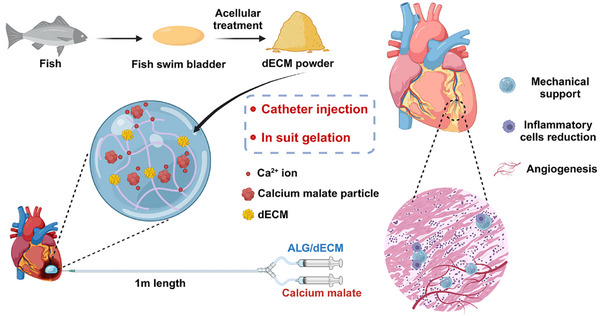
Schematic illustration of synthesis of Alg/dECM hydrogel and treatment for post‐MI cardiac repair. The cardiac function was improved due to the hydrogel treatment by improving mechanical support, inflammatory modulation and angiogenesis.

To develop an injectable hydrogel suitable for transcatheter intramyocardial injection, exhibiting exceptional reparative capabilities, we harnessed dECM derived from fish swim bladder to augment the bioactivity of alginate hydrogel. This enhancement was achieved by promoting vascularization and mitigating inflammation after MI. Furthermore, we used calcium malate, a slow‐release calcium ion reagent, as the crosslinker to ensure a gelation time conducive to transcatheter intramyocardial injection (**Figure** **1**). The data showed that the storage modulus (*G*′) after Alg@dECM mixed with the calcium ion crosslinker increased over time and remained steady over 1000 Pa after 5 min. In addition, the hydrogel could pass through a 1‐m transcatheter before gelation in the cardiac zone. In vitro assays proved that dECM increased angiogenesis by enhancing ECs’ behavior, such as tube formation, cell migration, and spreading, while modulating macrophage transition into the M2 phenotype. In a rat MI model, echocardiography on day 28 post‐MI demonstrated that the ejection fraction (EF) increased by ≈10% after hydrogel treatment, with increased angiogenesis, reduced inflammatory cells, alleviated infarct size, and improved left ventricular wall thickness (LVWD).

## 2 Results

### 2.1 The Protein Profiles of Fish Swim Bladder‐Derived ECMs

**Figure 2:**
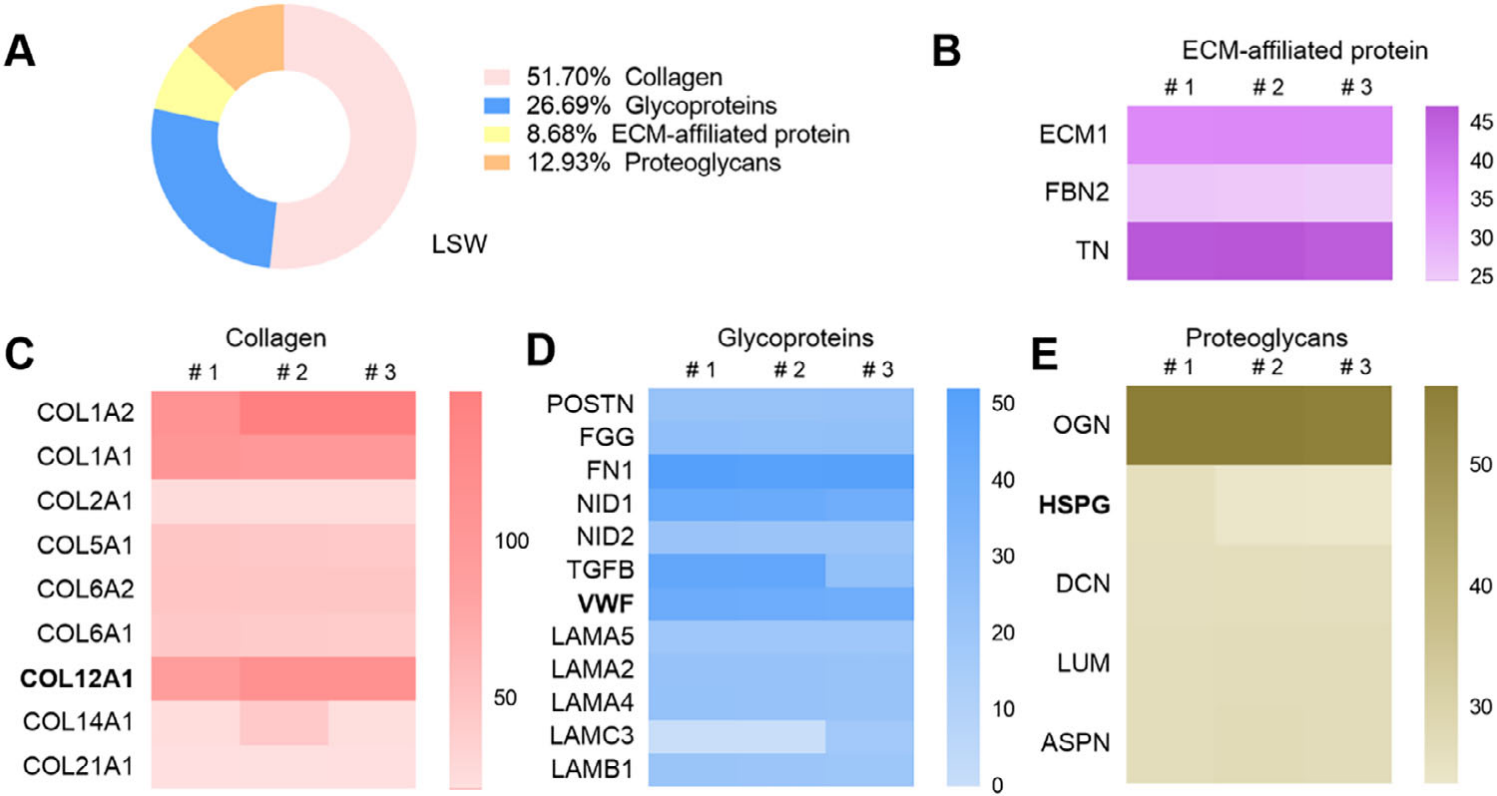
The protein profiles of fish swim bladder‐derived ECM were analyzed by liquid chromatography‐tandem mass spectrometry (LC‐MS/MS). A) The component of fish FSB‐extracellular matrix. B–E) The subtypes of ECM‐affiliated protein, collagen, glycoproteins, and proteoglycans.

The protein profiles of fish swim bladder‐derived ECMs were analyzed by liquid chromatography‐tandem Mass Spectrometry (LC‐MS/MS). Through protein identification and bioinformatic evaluation, 63 core extracellular matrix (ECM) proteins were elucidated. The results indicated that collagen, glycoproteins, and proteoglycans were the three major components (**Figure** **2**A,B), which account for 51.70%, 26.69%, and 12.93%, respectively. Label‐free quantification values were used to determine the relative abundance of matrix proteins. Among the collagens (Figure 2C), the genotypes with the highest relative abundance include COL1A1 (type I collagen), COL1A2, and COL12A1. The fish swim bladder is rich in type I collagen, which plays a crucial role in maintaining elasticity and mechanical strength.^[^ ^25^ ^]^ Glycoproteins include FN1 (fibronectin), LAMA (laminin), FGG (fibrinogen gamma), and LAMB (laminin beta 4). Key proteoglycans include OGN (osteoglycin), HSPG2 (heparan sulfate proteoglycan 2), DCN (Decorin), LUM (Lumican), and ASPN (Asporin). In addition, fish swim bladder materials contain the ECM affiliated proteins TNXB, ECM1, and FBN2. These extracellular matrix components not only provide mechanical support but also serve as sites for cell adhesion.^[^ ^26^ ^]^ Compared with the decellularized myocardial,^[^ ^24^ ^]^ there were 13 common proteins in FSB ECM, including Col1a1, Col1a2, Col5a1, Col6A1, FBN2, LAMA2, LAMA4, LAMB1, LUM, NID1, NID2, and POSTN. Furthermore, HSPG proteins were identified in FSB ECM, which are capable of covalently binding with cytokines to enhance repair functions. Third, Col12a1 and vWF were found in fish swim bladders, which are highly expressed in decellularized ECM from neonatal hearts but not the adult heart^[^ ^25^ ^]^ and contribute to heart regeneration.

### 2.2 Preparation and Characterization of Alg@dECM Hydrogel

**Figure 3:**
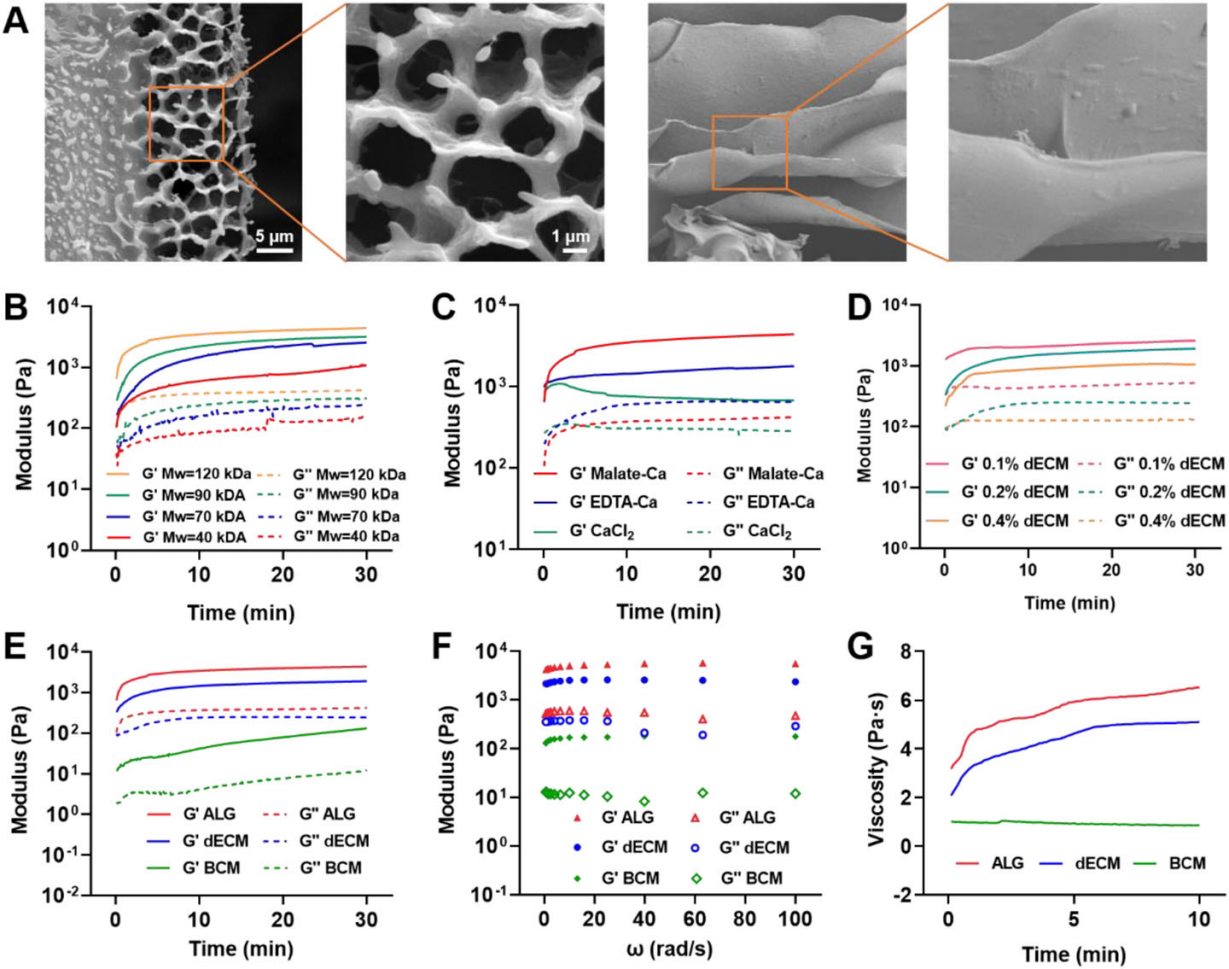
Preparation and characterization of Alg@dECM hydrogel. A) SEM images of ALG hydrogel, BCM hydrogel, and Alg@dECM hydrogel. B) Rheology of alginate hydrogels of different molecular weight. C) Rheology of alginate hydrogels crosslinked by different types of crosslinker agents. D) Rheology of alginate hydrogels incorporation with different contents of dECM. E) Rheology of ALG, BCM, and Alg@dECM hydrogels over time. F) Angular frequency. G) Viscosity of ALG, BCM, and Alg@dECM hydrogels over time.

To achieve an ideal hydrogel with suitable injectability and mechanical properties, various parameters were adjusted, including the molecular weight of alginate, solution concentration, types of crosslinker agents, and weight ratio of calcium ions to alginate (**Figure** **3**). The microstructures of ALG and ALG@dECM hydrogels were observed by SEM (Figure 3A). The ALG hydrogel after freeze‐drying exhibited a smooth sheet‐like microstructure. ALG@dECM possessed rougher surface and was branched with porous microstructures. These protrusions on surface and porous structures provided an ideal microenvironment for cell adhesion and migration. In terms of injectability, it was found that a mixture of 2% sodium alginate (Mw = 12 0kDa) with 1% calcium malate, or 0.3% CaCl_2_, or 0.4% EDTA‐Ca could be easily injected through a 27G needle, which is commonly used for human myocardium injection.^[^ ^27^ ^]^ Furthermore, the mechanical properties of the hydrogels were evaluated using oscillatory shear rheology (Figure 3B,C). The storage modulus *G*′ of the hydrogels enhanced as the molecular weight and concentration of alginate increased. For instance, when crosslinked by calcium malate, the *G*′ value increased from 1.0 to 4.4 kPa as the alginate molecular weight increased from 4 0to 12 0kDa (Figure 3B) and from 0.6 to 3.7 kPa as the concentration of alginate ranging from 0.5% to 2.0% (Figure S1, Supporting Information).

The gelation time is critical in selecting suitable hydrogel materials for myocardial injectable applications through transcatheter injection. Too fast gelling speed will cause blockage of the delivery catheter while too slow gelling speed will lead to colloid extrusion in the heart. Comparison among different crosslink agents revealed that the group crosslinked by calcium malate exhibited a suitable gelation time which took between 5 and 10 min compared to ≈2–3 min in CaCl_2_ group and ≈10 min in EDTA‐Ca Group (Figure 3C). In addition, in terms of mechanical properties, the group crosslinked by calcium malate had the higher storage modulus compared with the other groups. Thus, this hydrogel crosslinked by calcium malate was utilized in subsequent experiments and named ALG hydrogel.

To improve the effectiveness of ALG hydrogel, fish swim bladder ECM was added to improve cell compatibility (Figure 3D,E). The fish swim bladder was decellularized, freeze‐dried, and ground into a powder (Figure S2A, Supporting Information). Hematoxylin and eosin (H&E) staining confirmed decellularization, while Sirius Red and Verhöeff staining showed the composition of type I collagen and elastic fibers in the FSB dECMs (Figure S2B, Supporting Information). The mechanical properties of hydrogels increased with the incorporation content of FSB dECM from 0.1% to 0.4%. Based on cytocompatibility and mechanical properties, alginate hydrogel mixed with 0.2% dECM was used in subsequent experiments as ALG@dECM hydrogel. In comparison to BCM as a negative control, ALG@dECM and ALG groups had greater storage modulus values (1.9 and 4.4 kPa) compared to BCM group (0.1 kPa) (Figure 3F), indicating better mechanical support for tissues provided by these two types of hydrogels. The viscosities of each hydrogel were confirmed with shear rate of 1 Hz, showing that within 5 min after injection, the viscosity of the ALG@dECM hydrogel remained below 4 Pa s, making it injectable (Figure 3G).

Meanwhile, the swelling ratio and rheological property of ALG and ALG@dECM hydrogels in both dry and hydrous status after swelling were evaluated. As shown in support Figure 3A, D4, in different states all the hydrogels showed good anti‐swelling performance when immersed in PBS, and could reach swelling equilibrium within 90 min. And the swelling ratio of alginate hydrogel decreased with the added of dECM (2043.9% in dry state and 81.3% in hydrous status). Rheological characterization indicated that after swelling, the mechanical properties of hydrogels remain unchanged in dry state, but the *G*′ of ALG@dECM decreased in hydrous status (Figure 3B,E, Supporting Information). Although the mechanical properties has changed, the ALG@dECM hydrogel remained a relatively stable state (*G*′ > *G*″) (Figure 3C,F).

### 2.3 The Mechanical Stability of Hydrogels

**Figure 4:**
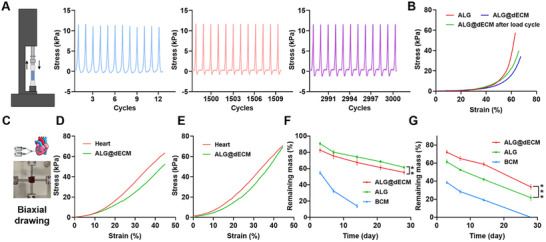
Mechanical stability and degradation of ALG@dECM hydrogel. A) Load cycle curve of compression 3000 cycles of the ALG@dECM hydrogel during 1–10, 1500–1510, and 2990–3000 cycles. B) Compress stress–strain curves of ALG, dECM, and ALG@dECM hydrogels. C) Schematic diagram of planar biaxial mechanical tests of ALG@dECM hydrogel after heart injection. D,E) Tension stress–strain curves of hydrogel‐embedded tissue in different axis. F) In vitro degradation of ALG, BCM, and Alg@dECM hydrogels in vitro assay. G) In vivo degradation of ALG, BCM, and Alg@dECM hydrogels in a mice subcutaneous implantation model (*n* = 5). All data in the figure are presented as mean ± SEM (*p < 0.05, **p < 0.01, and ***p < 0.001).

The good fatigue resistance is essential for hydrogels to maintain their function in myocardium repair. And materials with appropriate elasticity can be used to synchronize cardiomyocyte contraction in vitro and to steadily support cardiac systole and diastole in vivo. The ALG@dECM hydrogel was subjected to 3000 cycles of loading–unloading compression tests at 50% strain. **Figure** **4**A displayed that after 3000 cycles, the hydrogel still maintained its original elastic modulus. In addition, after repeated compression, the compression curves of the hydrogel did not alter too much compared to the freshly prepared hydrogel (Figure 4B), indicating that the hydrogel could offer durable and consistent support for cardiac contraction. To investigate the changes in mechanical properties of cardiac tissue after hydrogel injection, we injected the ALG@dECM hydrogel into the left ventricle of porcine heart through a 1‐m transcatheter, then we extracted the hydrogel‐embedded tissue with an adjacent tissue from the same cross‐section to carry out planar biaxial mechanical tests (Figure 4C). The results showed that the mechanical properties of cardiac tissues remained stable after hydrogel injection (Figure 4D,E).

### 2.4 The Degradation and Safety of ALG@dECM Hydrogel

It is critical for implant materials to degrade and be metabolized in the suitable time after supporting heart tissue repair and regeneration. In vitro assay (Figure 4F) showed that the ALG and ALG@dECM hydrogels left more than 55% of their original mass, while the BCM hydrogel had completely degraded at 28 d after incubation in PBS solution. In vivo assay (Figure 4G) by a mice subcutaneous implantation model (Figure S4, Supporting Information) showed that the weight of ALG@dECM hydrogel remained 58% on day 14 and 34% on day 28, which maintained significantly longer than BCM and ALG hydrogels. It probably owes to the cell infiltration and extracellular matrix formation in the ALG@dECM hydrogel. In addition, histopathologic analysis by H&E staining (Figure S5,S6, Supporting Information) proved there were no obvious changes in the main organs of mice, suggesting that the ALG@dECM hydrogel was nontoxicity.

### 2.5 Cytocompatibility of ALG@dECM Hydrogel on Myocytes

**Figure 5:**
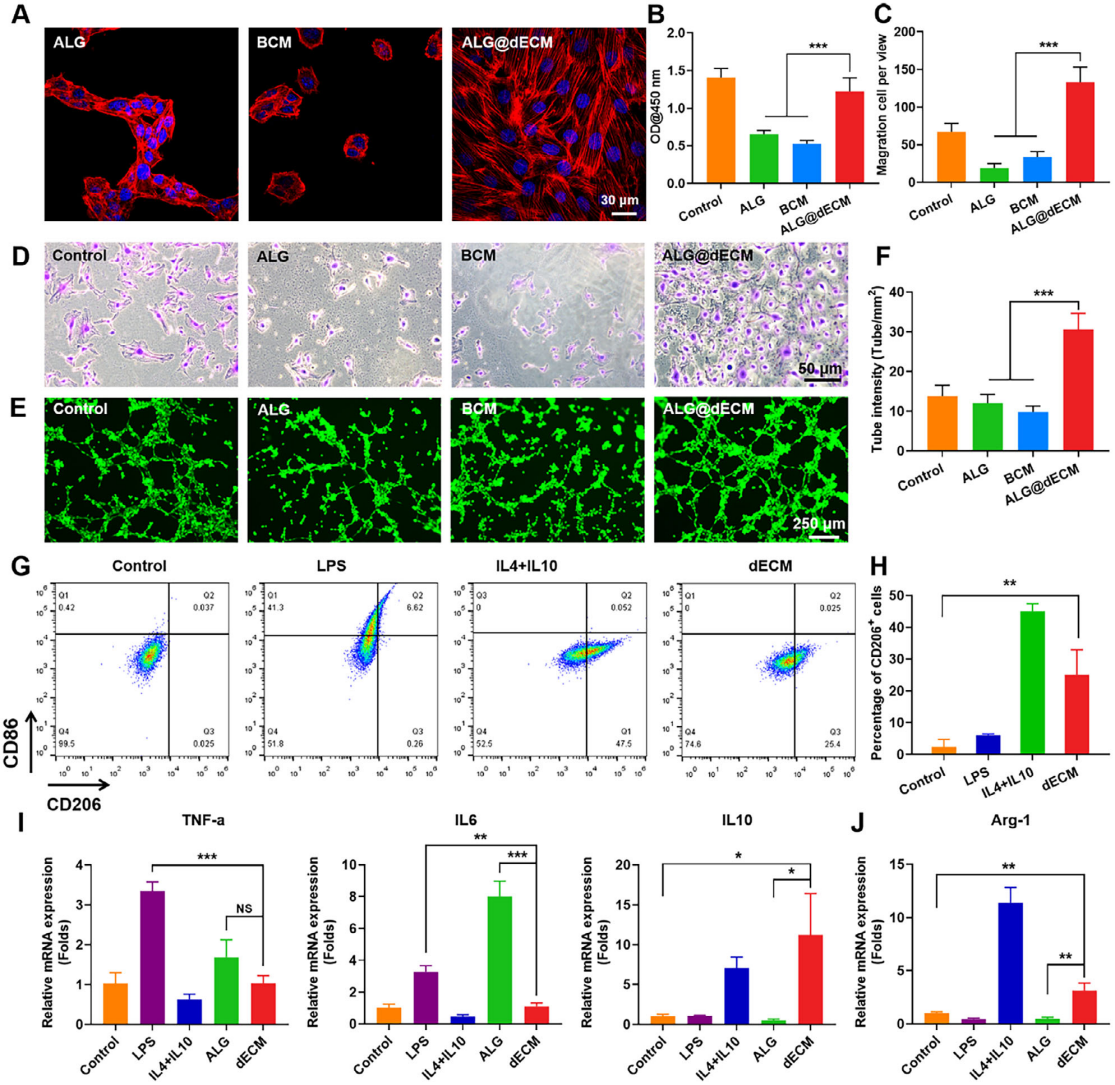
ALG@dECM hydrogel promoted cells viability, migration, and tube formation. A) The F‐actin stained of cytoskeleton of H9C2 cells on different hydrogel. B) The OD values of H9C2 cells cultured for 2 d in ALG, dECM, and Alg@dECM hydrogels by CCK8 assay. C) Images and quantitative analysis of transwell assay for HUVECs at 24 h (*n* = 5). D) Images and quantitative analysis of tube formation of HUVECs (*n* = 5). G–J) Fish swim bladder dECM promoted the macrophages polarization into an anti‐inflammatory M2 phenotype (*n* = 3). G,H) Proportion of CD206^+^ and CD86^+^ expression cells in F4/80^+^ after RAW267.4 cells seeded on the hydrogels. I,J) Relative mRNA expression of TNF‐α, IL‐6, IL‐10, and Arg‐1 (*n* = 3). All data in the figure are presented as mean ± SEM (*p < 0.05, **p < 0.01, and ***p < 0.001).

The cytocompatibility was tested by seeding H9C2 cells (a subclone of the original clonal cell line derived from embryonic BD1X rat heart tissue) on BCM, ALG, and ALG@dECM hydrogels. Considering that the dECM from fish swim bladder can promote cells adhesion and stretching, we stained the H9C2 cells cultured on the hydrogels with F‐actin antibody. As shown in **Figure** **5**A, cells seeded on the ALG@dECM hydrogel extended and adhered well, while the morphology of cells on BCM and ALG hydrogels remained round, indicating that the ALG@dECM hydrogel had better biocompatibility than Alg and BCM. After 2 d, cell counting kit‐8 (CCK‐8) results showed that the cell viability of H9C2 cells was significantly higher than that of BCM and ALG hydrogels (Figure 5B).

### 2.6 Migration and Tube Formation of Endothelial Cells on ALG@dECM Hydrogel

Migration of endothelial cells greatly promotes vascular regeneration, which is necessary for tissue regeneration.^[^ ^28^ ^]^ Wound healing and transwell assay were performed to evaluate human umbilical vein endothelial cells (HUVECs) migration on hydrogel. Representative images of wound scratch assay can be seen in Figure S6H (Supporting Information). HUVECs treated with ALG@dECM hydrogel migrated (migration ratio of 80%) to the cell‐free area faster than ALG (68%) and BCM (70%) groups. Meanwhile, the transwell assay (Figure 5C,D) demonstrated that the ALG@dECM hydrogel could attract more HUVECs to pass through the pores of the upper chamber and migrate to the lower chamber. Next, the proangiogenic effect of ALG@dECM hydrogel was evaluated by tube formation assay (Figure 5E,F). After 4 h of incubation, the cells cultured on ALG@dECM hydrogel formed higher tube density and more junctions than other groups. These results showed that the ALG@dECM hydrogel could effectively promote tube formation and migration of endothelial cells, which may facilitate the revascularization of the injured cardiac tissues.

### 2.7 Macrophage Polarization by ALG@dECM Hydrogel

Macrophages are important for myocardial repair, in which M2 macrophages have anti‐inflammatory effects and promote angiogenesis and tissue repair.^[^ ^29^, ^30^ ^]^ However, the presence of macrophages is associated with the deposition of collagen fibers. According to the previous studies,^[^ ^31^ ^]^ ECM extract from small intestinal submucosa could promote the constructive M2 macrophage phenotype. Therefore, we assumed that ECM from fish swim bladder could regulate macrophage polarization towards M2 phenotype. Because it is difficult to completely digest cells planted on hydrogel, we applied ECM solution to stimulate RAW 264.7 cells (a macrophage cell line established from mouse tumor) in vitro and analyze the cell phenotype using flow cytometry (**Figure** 5G,H). Lipopolysaccharide (LPS), interleukin‐4 (IL‐4), and interleukin‐10 (IL‐10), were also used to stimulate macrophages as controls. In Figure 5G,H, treatment with the dECM could promote the polarization of macrophages to an anti‐inflammatory M2 phenotype (mean percentage 25%). The data also indicated that dECM induced only a minimal number of M1 macrophages, which suggests that it does not cause an inflammatory response.

**Figure 6:**
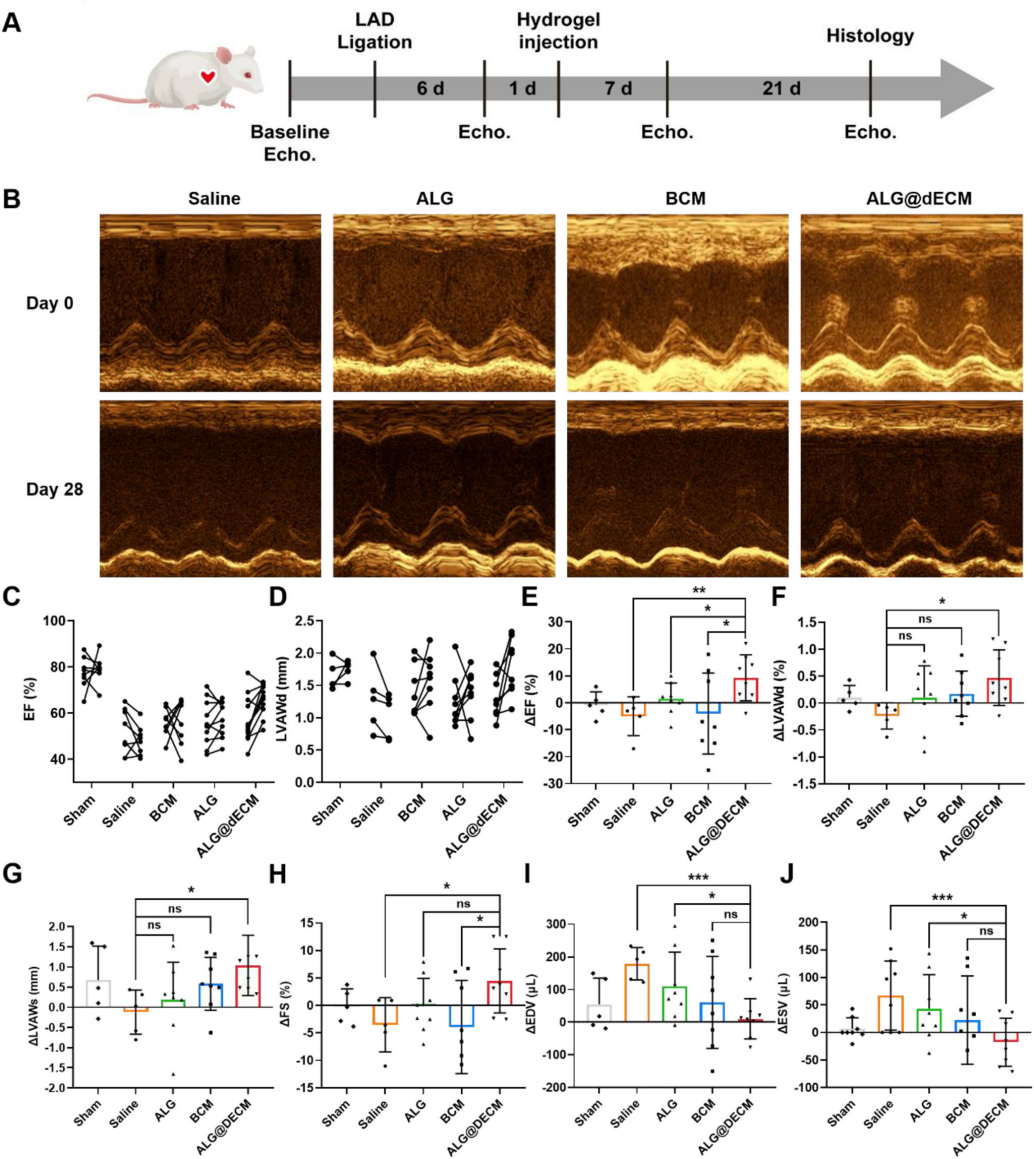
ALG@dECM hydrogel improved cardiac function in rat MI model. A) Schematic diagram of treatment HF in a rat MI model. B) Representative M‐mode echocardiographic images of heart function. C,E) EF values and its variation of left ventricular in different groups (Sham group, *n* = 5; Saline group, *n* = 5; ALG group, *n* = 8; BCM group, *n* = 8; ALG@dECM group, *n* = 8). D,F) LVAWds and its variation of left ventricular in different groups. G–J) Variation of LVAWs, FS, EDV, and ESV in different groups. All data in the figure are presented as mean ± SEM (*p < 0.05, **p < 0.01, and ***p < 0.001).

Subsequently, the RAW 264.7 cells were cultivated in the ALG and ALG@dECM hydrogels for 24 h. Following incubation, the cells encapsulated in the hydrogels were released using 10 mg mL^−1^ sodium citrate.^[^ ^32^ ^]^ Next, total messenger RNA was extracted from cells with TRIzol reagent, and reverse transcription polymerase chain reaction (RT‐PCR) was used to detect the expression of genes associated with macrophages, with TNF‐α, IL‐6 denoting M1 cell‐associated gene and IL‐10, ARG‐1 denoting M2 cell‐associated gene. Figure 5I illustrates that M1 macrophage related genes are markedly expressed in the ALG group, while M2 macrophage‐ related genes are highly expressed in the ALG@dECM group. This result may be attributed to the fact that the high Ca^2+^ environment in alginate hydrogel drives inflammation^[^ ^33^ ^]^ and dECM in hydrogel inhibits this trend.

### 2.8 Antioxidant Activity of ALG@dECM Hydrogel in Rat Primary Cardiomyocytes

Excessive ROS production is considered to be a substantial reason for MI development. The ROS scavenging capacity of hydrogels was investigated in rat primary cardiomyocytes by DCFH‐DA. In physiological conditions, the degradation products of alginate(β‐d‐mannuronic and α‐l‐guluronic) exhibit good antioxidant activity, and cysteine contained in FSB also possesses antioxidant capabilities. Therefore, compared to the H_2_O_2_ group, the ROS level was reduced in the hydrogels treatment group after 24 h, with the ALG@dECM group showing the greatest reduction (Figure S7A,B, Supporting Information).

### 2.9 Therapeutic Effects of ALG@dECM Hydrogel in Rat MI Model

Figure 6B displays representative M‐mode echocardiographic images captured at the mid‐papillary level of the left ventricular short axis. At baseline, no notable disparities were observed in any of the parameters across the BCM, ALG, dECM, and Saline groups. Following injection, remarkable temporal variations in EF and FS were evident in the BCM, ALG, and dECM. Notably, the ALG@dECM group demonstrated a substantial enhancement of 9.3% in EF and 4.5% in FS, whereas the other groups remained unaltered (Figure 6E,H). Moreover, the ALG@dECM group experienced a pronounced increase of ≈0.47 mm in LVAWd from day 0 to day 28, surpassing the modest gains in the BCM (0.10 mm) and ALG (0.18 mm) groups (Figure 6F). In addition, the Alg@dECM group showed dramatically decreased difference value of end‐diastolic volume (ΔEDV) and end‐systolic volume (ΔESV), compared to Saline and Alg groups (Figure 6I,J). These data together indicate that the hydrogel can reduce wall stress and modify cardiac function by increasing wall thickness and stabilizing chamber dimension.

**Figure 7:**
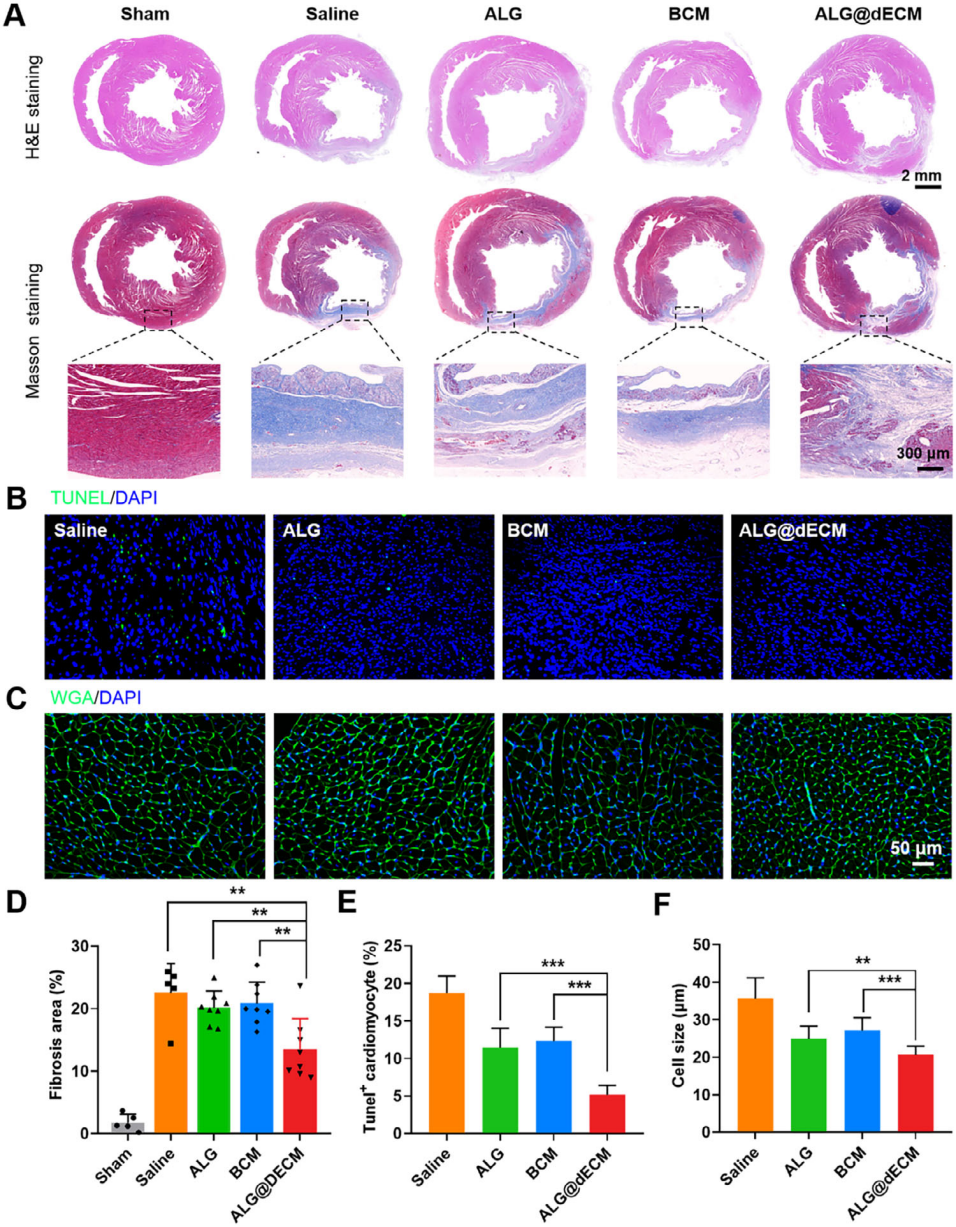
Therapeutic effects of ALG@dECM hydrogel in rat MI model. A) H&E and Masson trichrome staining of harvested hearts from rats. B,E) Representative images and quantitative analysis of TUNEL staining in infarct area (*n* = 5). C,F) Representative images and quantitative analysis of WGA immunofluorescence staining in the border area (*n* = 5). D) Quantification of infarct area by Masson staining (Sham group, *n* = 5; Saline group, *n* = 5; ALG group, *n* = 8; BCM group, *n* = 8; ALG@dECM group, *n* = 8). All data in the figure are presented as mean ± SEM (*p < 0.05, **p < 0.01, and ***p < 0.001).

Histological assessments (**Figure** **7**A) revealed collagen deposition across all MI models, yet ALG@dECM therapy notably diminished the infarcted area by 13.5% (Figure 7D), in contrast to the ALG (20.19%) and BCM (20.86%) groups, which did not significantly differ from the saline control (22.57%). In addition, an augmentation in left ventricular wall thickness was observed in both ALG and ALG@dECM‐treated animals (Figure S8, Supporting Information), suggesting that hydrogels with greater stiffness might offer superior mechanical reinforcement to damaged cardiac tissues. The loss of CMs and their decompensated hypertrophy are pivotal factors contributing to cardiac remodeling and the progression of heart failure after myocardial infarction. To assess CM apoptosis, TUNEL staining (Figure 7B,E) was used, revealing that the ALG@dECM hydrogel effectively mitigated the apoptotic burden in infarcted myocardial tissue. To evaluate CM hypertrophy in the infarcted hearts (Figure 7C,F), WGA staining was utilized. Notably, after a period of four weeks, the cross‐sectional areas of CMs were significantly enlarged in the saline and BCM groups, whereas these changes were absent in the ALG and ALG@dECM groups, suggesting the potential of ALG and ALG@dECM in restoring cardiac function. In addition, there is a decreasing trend in heart weight / body weight of ALG@dECM group (Figure S9, Supporting Information).

**Figure 8:**
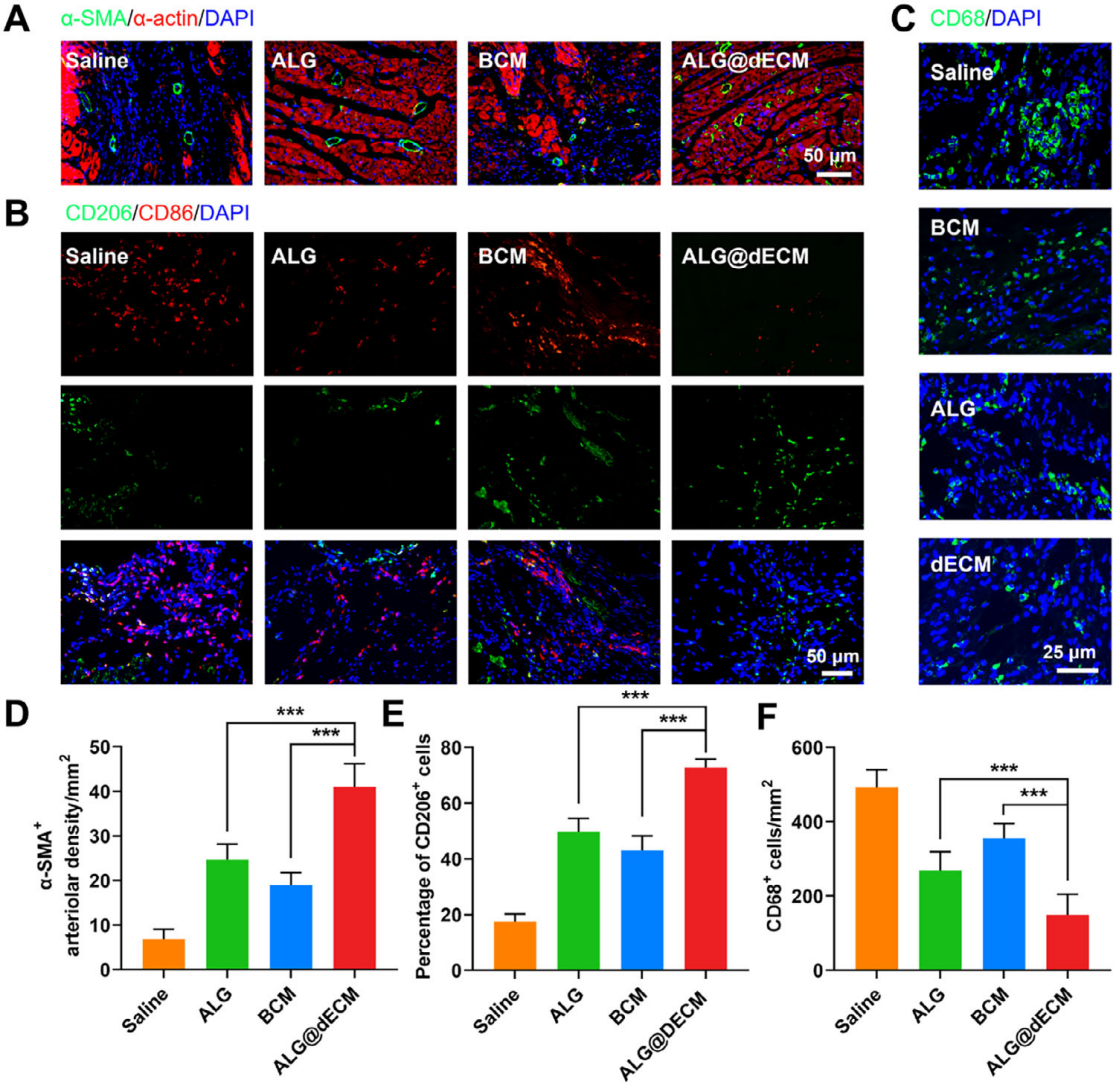
The therapeutic mechanism of ALG@dECM hydrogel in rat MI model. A,D) Representative images and quantitative analysis of α‐SMA immunofluorescence staining in infarct area (*n* = 5). B,E) Representative images of CD86/CD206 immunofluorescence staining in infarct area and statistics of the proportion of M2 macrophages (*n* = 5). C,F) Representative images and quantitative analysis of CD68 immunofluorescence staining in infarct area (*n* = 5). All data in the figure are presented as mean ± SEM (*p < 0.05, **p < 0.01, and ***p < 0.001).

Furthermore, the ALG, BCM, and ALG@dECM groups exhibited significantly elevated arteriolar densities compared to the Saline group (**Figure** **8**A,D). Notably, the ALG@dECM group demonstrated the highest vessel density, accompanied by a pronounced presence of α‐actinin‐positive cells surrounding these vessels. This observation suggests that the ALG@dECM hydrogel has the potential to stimulate angiogenesis, thereby favorably promote the survival of cardiomyocytes. To investigate the underlying mechanism of ALG@dECM hydrogel in facilitating cardiac healing, we further evaluated the counts and proportions of macrophages throughout the inflammatory response phase (Figure 8B,E). Our analysis revealed a notably decreased density of CD68+ macrophages in the infarct zone of hearts treated with ALG@dECM hydrogel, as compared to those subjected to saline, BCM, and ALG treatments alone. Furthermore, immunofluorescence staining illuminated a compelling trend, with the ALG@dECM‐treated group exhibiting a significantly higher prevalence of M2 macrophages (76%) within the infarct area, surpassing both ALG (51%) and BCM (47%) treatment groups (Figure 8C,F). These findings strongly suggest that the integration of fish swim bladder ECM into the hydrogel formulation not only mitigates inflammation but also fosters the polarization of macrophages towards the M2 phenotype, thereby facilitating myocardial tissue regeneration and repair.

### 2.10 Transcriptome RNA Sequencing Compared to the Effects of Different Hydrogels on the Gene Level of Left Ventricle Tissues in the Infarct Heart

**Figure 9:**
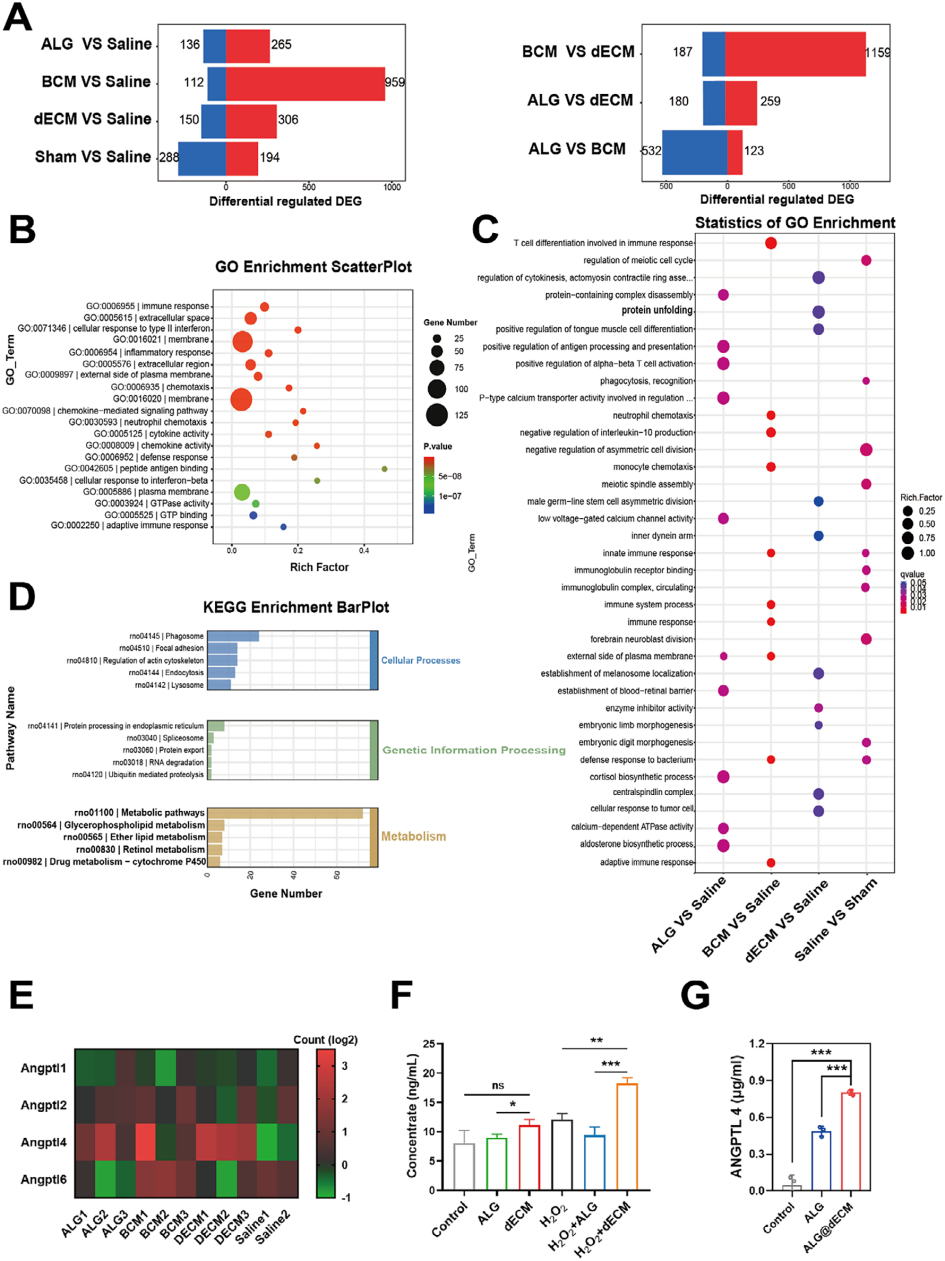
Transcriptome RNA sequencing of MI rats in different groups at 7 d after hydrogel injection. A) Differentially expressed genes (DEGs) in each pairwise comparison. Red represents the number of upregulated genes, and blue represents the number of downregulated genes. B) GO enrichment scatterplot based on differential genes comparing ALG and dECM group. C) GO enrichment scatterplot based on differential genes comparing the groups (ALG vs Saline, BCM vs Saline, dECM vs Saline, and Saline vs Sham). D) KEGG enrichment barplot shows the pathway of interest in the DEGs when compared across multiple groups (ALG, BCM, dECM, Saline, Sham). The *x*‐axis represents the number of DEGs within a pathway, the *y*‐axis indicates the pathway names, and the color denotes the KEGG primary classification. E) Heatmap of Angptl4 family proteins across all samples. F) ANGPTL4 expression in H9C2 cells before and after dECM treatment by Elisa assay. G) The adsorption of ANGPTL4 in Alg and Alg/dECM hydrogel in vitro assay.

To further elucidate the mechanisms underlying the effects of ALG, BCM, and dECM hydrogels on myocardial infarction in rats, we conducted whole transcriptome RNA sequencing using the collection of cardiac tissue from the apical region of 7 d post‐treatment MI rats, alongside sham controls. Initially, we analyzed the differentially expressed genes (DEGs) among the treatment groups compared to the saline group, as well as between the different hydrogel treatments. Our statistical analysis indicated that, hydrogels exerted distinct influences on the upregulation and downregulation of genes in cardiac tissue, with the BCM group exhibiting a higher number of upregulated genes compared to the ALG and dECM groups (**Figure** **9**A). Furthermore, to specifically assess the impact of fish bladder acellular matrix (dECM) on gene expression in cardiac tissue, we performed Gene Ontology (GO) analysis on the DEGs identified between the ALG and dECM groups. The dECM significantly affected gene functions associated with immune response, extracellular matrix organization, cellular response to type II interferon, membrane, and inflammatory responses (Figure 9B). Subsequent GO analysis of DEGs among all groups, including the saline group, revealed that dECM had a pronounced effect on processes such as actomyosin ring assembly, protein unfolding, and enzyme inhibitor activity. Notably, the BCM hydrogel was found to influence a larger number of genes related to immune responses, while ALG predominantly affected genes involved in calcium dependent ATPase activity and the positive regulation of alpha‐beta T cell activation (Figure 9C). Additionally, KEGG pathway analysis of the DEGs across the five groups identified key pathways impacted by differential gene expression, including the phagosome, protein processing in endoplasmic reticulum, and metabolic pathways (Figure 9D). From bulk RNAseq analysis, protein unfolding signaling in GO enrichment and metabolic pathways in KEGG enrichment were highly expressed in the ECM Group. ANGPTL4 was identified as the only common protein in both pathways. Notably, ANGPTL4 is uniquely expressed in the ECM group within the ANGPTL family proteins (Figure 9E). In a line, dECM treatment significantly elevated ANGPTL4 expression in H9C2 cells, particularly under H2O2 pre‐treatment conditions (Figure 9F) in in vitro assay. In addition, we also observed that dECM enhanced ANGPTL4 protein adsorption into hydrogel (Figure 9G). As far as we know, ANGPTL4 is involved in lipid metabolism and participant in regulating cardiomyocytes. Specifically, increased ANGPTL4 inhibits LPL enzyme activity, reducing fatty acid (FA) production and alleviating FA in cardiomyocytes and finally preventing fat toxicity and oxidative stress in cardiomyocyte.

## 3 Discussion

In this study, we developed a transcatheter injectable fish swim bladder‐based hydrogel demonstrating excellent cell compatibility and mechanical properties. Calcium malate cross‐linked alginate hydrogel combined with dECM extracted from swim bladder shows effective repair of myocardial infarction injury and protection of post‐MI cardiac function. ALG@dECM hydrogel exhibited stable mechanical properties in loading–unloading compression tests and planar biaxial mechanical tests, providing lasting and consistent support for heart contractions. In vivo experiments demonstrated that the injection of ALG@dECM hydrogel into the infarcted area in rats resulted in more pronounced left ventricular anterior wall motion, increased ejection fraction, and increased anterior wall thickness, thus protecting heart function after infarction.

The delivery method of injectable hydrogel has a significant impact on the efficacy and safety of hydrogel therapy. However, it is often ignored in biomaterials design and fabrication. Compare to the direct epicardial injection and intracoronary delivery, transcatheter‐based delivery approach seems a much more translationally relevant method, due to its low risks and quick recovery times. For catheter delivery, there is unique design criteria for injectable materials, distinct from other injectable hydrogels.^[^ ^34^ ^]^ The materials must have appropriate gelation kinetics to travel through a long, small‐diameter catheter (typically 26‐gauge) and gel in the infarct, but not in the catheter.^[^ ^23^ ^]^ In this study, a hybrid alginate@ECM hydrogel suitable for cardiac stabilization via transcatheter endomyocardial injection was successfully developed. To optimize the rheological properties of alginate hydrogel, we used calcium malate as a crosslinker, which could slow release Ca^2+^, rather than CaCl_2_, or bioglass_3_, typically used, which cause fast gelation in the outside while still liquid inside, resulting in uniform mechanical properties and uncontrolled the gelation time.^[^ ^35^, ^36^ ^]^ And our innovative formulate takes about 5–6 min for gelation, which can guarantee that the Alg/ECM could easily pass through the delivered catheter and gel in the heart zone. In addition, the mechanical strength and stability of hydrogels are also important. As literature reports, the storage modulus of ideal injectable biomaterials should be ≈1000 Pa,^[^ ^37^, ^38^ ^]^ by providing mechanical support of damaged tissue and suitable for cell infiltration. Hydrogel with too stiffness usually result in abnormal heart muscle movement in diastolic and systolic, and also hinder cell infiltration and proliferation, such as alginate alone (3–5 kPa).^[^ ^39^ ^]^ Meanwhile, hydrogel with low stiffness cannot provide enough mechanical support, such as ECM alone with a very low storage modulus about 5 Pa.^[^ ^40^ ^]^ Herein, storage modulus of our Alg@ECM was about 1000 Pa and the compressive modulus could maintain stable even after 3000 cycles. In combined with slow degradation, make this hydrogel exert mechanical support for a long time, resulting in reversal of the remodeling process of heart after injury.

To understand the mechanism how Alg@ECM hydrogels for MI treatment is of great significance for its clinical translation. Herein, we consider Alg@dECM hydrogel exert multiple roles in cardiac repair, which can be divided into three sequential stages. Initially, the hydrogel functions predominantly as a filler and a support structure, providing crucial mechanical support. This mechanical support is fundamental for maintaining the structural integrity of the myocardial tissue. It helps to physically reinforce the damaged area, preventing further tissue deterioration and providing a stable framework for subsequent cellular activities. In the second stage, Alg@dECM hydrogels assume a regulatory role, primarily attributed to the ECM it contains. The FSB‐ECM is composed of a complex mixture of biomolecules, such as HSPG and Col12a1 (Figure 2). These components actively participate in modulating the immune response, ensuring that the body's immune system responds appropriately to the injury without causing excessive inflammation that could exacerbate the damage. Simultaneously, the ECM promotes angiogenesis, the formation of new blood vessels. This is essential for restoring blood supply to the damaged myocardium, delivering oxygen and nutrients necessary for tissue repair and regeneration. Our in vitro experiments demonstrated that compared to ALG and BCM, ALG@dECM exhibited superior promotion of HUVECs migration and tube formation. In MI rats, immunofluorescence staining results from the application of ALG@dECM revealed that the density of newly formed arterioles marked by α‐SMA was significantly higher than that in the saline group and surpassed that in both the ALG and BCM groups. These results indicate that dECM contributes to the survival and tissue reconstruction of vascular endothelial cells and smooth muscle cells in the local ischemic and hypoxic environments post‐MI. Moreover, our findings revealed that ALG@dECM can inhibit macrophage aggregation in the infarct area and increase the proportion of M2 macrophages with repair functions. ALG@dECM reduced the number of macrophages in the infarct area post‐myocardial infarction, leading to a higher proportion of M2 macrophages. This indicates that one protective effect of ALG@dECM on the myocardium is the prevention of excessive cardiac fibrosis post‐myocardial infarction by regulating macrophage recruitment and M1/M2 macrophage differentiation. During the third stage, the ECM plays a pivotal role in regulating myocardial energy metabolism. By modulating key metabolic pathways, it enables the cardiomyocytes to efficiently utilize energy sources, enhancing their survival and functionality. In the present study, we also discovered that the FSB‐ECM has the capacity to upregulate the expression of ANGPTL4, a protein with multifaceted functions in fatty acid metabolism, angiogenesis, and inflammation regulation. Leveraging the RNA‐seq pathway enrichment analysis carried out in this project, we hypothesized that the ECM activates ANGPTL4 to mediate the regulation of myocardial metabolism. This activation sets in motion a series of biological processes that ultimately promote myocardial repair. For example, enhanced fatty acid metabolism provides an alternative energy substrate for the stressed cardiomyocytes, while the promotion of angiogenesis improves tissue perfusion, and the regulation of inflammation creates a conducive microenvironment for tissue regeneration. However, the in depth of how ANGPTL4 modulates this repair process is needed further investigation.

In summary, the Alg@dECM has emerged as a multi‐effect factor profoundly influencing inflammation regulation, angiogenesis, myocardial metabolism, and native myocardial regeneration. Its complex functions underscore its significance as a potential therapeutic target for cardiac repair and regenerative medicine. The ability of ALG@dECM to promote angiogenesis, regulate macrophage behavior, and potentially activate ANGPTL4‐mediated metabolic regulation further validates the importance of hydrogel‐based ECM in treating myocardial infarction.

## 4 Experimental Section

### Materials

Sodium alginate (Mw: 40000–120000, α‐l‐guluronic/β‐d‐mannuronic = 2:1) was purchased from Haizhilin (China). Calcium malate was obtained from Macklin (China). FITC‐conjugated wheat germ agglutinin was purchased from Sigma (Germany). Sodium dodecyl sulfate (SDS), 2‐[4‐(2,4,4‐trimethylpentan‐2‐yl) phenoxy] ethanol (Triton X‐100) and cell counting kit‐8 were purchased from Solarbio (China). DNase and RNase were purchased from CWBIO (China). LPS was obtained from Sigma (L2880). IL‐4 (574302), IL‐10(575804), and the flow cytometry antibody APC anti‐mouse F4/80 (123115), FITC anti‐mouse CD206 (141703), and PE anti‐mouse CD86 (159203) were purchased from biolegend (USA). The primary antibodies against F‐actin (ab205), CD68 (ab15212), CD86 (ab220188), CD206 (ab64693), α‐SMA (ab7817) and the secondary antibodies Alexa Fluor 488‐conjugated Goat Anti‐Rabbit IgG H&L(ab150077), Alexa Fluor 594‐conjugated Goat Anti‐Rabbit IgG H&L (ab150080), and Alexa Fluor 594‐conjugated Goat Anti‐Mouse IgG H&L (150120) were ordered from Abcam. The primary antibody against CD86 (sc‐28347) was obtained from Santa Cruz. All animal procedures in this study were approved by the Peking Union Medical College Hospital's Animal Experimentation Ethics Committee.

### Preparation of Fish Swim Bladder dECM

First, the swim bladder was washed off surface blood stains and mucous membranes, cut into squares with sides of about 4 cm, and placed in 1% SDS solution at room temperature for 6 h. The tissue was then washed several times with sterile PBS and treated with 1% 2‐[4‐(2,4,4‐trimethylpentan‐2‐yl) phenoxy] ethanol (Triton X‐100) solution for 0.5 h at room temperature. Next, the swim bladder tissue was washed several times with sterile PBS and then washed in freshly made sterile PBS for one week until the solution was free of foam. Finally, the swim bladder was immersed in 2 U mL^−1^ DNase with 0.1 mg mL^−1^ RNase buffer and treated with constant temperature shaking at 37 °C overnight. The decellularized tissue was frozen at −20 °C, freeze‐dried and ground into powder. The protein profiles of fish swim bladder‐derived ECM were analyzed by liquid chromatography‐tandem mass spectrometry (LC‐MS/MS).

### Fabrication of Different Hydrogels

For selection of calcium‐crosslinked alginate solution, several parameters such as alginate molecular weight, the type and weight ratio of calcium crosslinker were adjusted. Then the sodium alginate solution and calcium ions solution were mixed with a three‐way valve and injected through 27G needle to yield a calcium‐crosslinked alginate hydrogel displaying low apparent viscosity.

For ALG@dECM hydrogel, the matrix powder was then mixed with sodium alginate at a mass ratio of 0.5:10, 1:10, and 2:10 (dECM/ALG), dissolved in deionized water, and made the final concentration of sodium alginate to be 2%. The solution was mixed with a 1% calcium malate suspension to obtain the ALG@dECM hydrogel.

### Characterizations of Different Hydrogels

Rheological properties of BCM, ALG, and ALG@dECM hydrogels were measured by rheometer (MCR302, Anton Paar, Austria). Briefly, the test was conducted at 37 °C in oscillation mode with a frequency of 1Hz. Then 1 mL of each hydrogel was tested and the relevant data was collected in 30 min after injection. For microstructures observation, the prepared hydrogels were lyophilized and its morphologies were observed by scanning electron microscope (SEM, GeminiSEM 360, ZEISS, Germany).

For swelling ratio test, 500 µL of hydrogels (*W*_0_) were added to 2 mL of PBS. The hydrogels were removed from PBS every 10 min, and their weights (*W*_t_) were measured after surface moisture wiped off. Finally, the swelling ratio was calculated by the following formula

(1) $$\mathrm{Swelling}\;\;\mathrm{ratio}=\frac{W_{t}-W_{0}}{W_{0}}$$

For mechanical properties test, all hydrogels were cut into a cube with a side length of 1 cm. The mechanical properties were then detected by universal testing machine (3355, Instron, USA) at a compression speed of 600 mm min^−1^. The cardiac tissues were cut into a 60 mm × 60 mm × 5 mm rectangular. After that, the biaxial tensile test of cardiac tissues was conducted by biaxial tensile tester (Kaier, China).

### Cytotoxicity Test and Fluorescent Immunoassay of H9C2 Cells on Different Hydrogels

To evaluate the cytotoxicity of BCM, ALG, and ALG@dECM hydrogel, CCK‐8 test was carried out on H9C2 cells loaded on the hydrogels. For fluorescent immunoassay, H9C2 cells were cultivated on different hydrogels for 2 d, then all the samples were incubated in primary antibodies against F‐actin (1:500) overnight at 4 °C, rinsed with PBS, and incubated with Alexa Fluor 594‐conjugated Goat Anti‐Mouse IgG H&L (1:1000) secondary antibodies for 1 h at RT. The stained samples were further incubated with 4′,6‐diamidino‐2‐phenylindole (DAPI) for 30 min and observed under confocal laser scanning microscope (LSM710, ZEISS, Germany).

### Wound Healing Test, Transwell Assay, and Tube Formation Assay

*Wound Healing Test*: HUVECs were seeded at a density of 1.5 × 10^5^/well in a six‐well plate and cultured to cover the well at 37 °C. The cells were scratched with 200 µL pipette tips and washed with PBS three times to remove the suspension cells. Then 1 mL serum free medium and 1 mL hydrogels were added into the well. The cells were recorded at 0 and 12 h by photography (DMi8, Leica, Germany).

*Transwell Assay*: HUVECs were seeded at a density of 5 × 10^4^/well in the upper chamber of a 24‐well plate. The lower chamber was filled with 400 µL serum‐free culture medium and 100 µL hydrogels. After incubation for 24 h, the migrated cells on the lower chamber were stained with crystal violet solution (0.5%) for 30 min and observed under the inverted microscope (DMi8, Leica, Germany).

*Tube Formation Assay*: Matrigel (354248, Corning, USA) was diluted with hydrogels in a volume ratio of 1:1. Then 50 µL mixed hydrogel was added to a precooled 96‐well plate and incubated for 30 min at 37 °C. After that, 50 µL cell culture medium containing 1 × 10^4^ HUVECs were seeded and further incubated for 4 h. HUVECs were stained with calcein solution and observed under the inverted microscope (DMi8, Leica, Germany). Finally, the tube density was calculated by ImageJ software.

### Flow Cytometry

RAW246.7 (a macrophage cell line that was established from a tumor in a male mouse) cells were planted at a density of 1 × 10^7^/well of a six‐well plate. And the cells were treated with 100 ng mL^−1^ LPS, 20 ng mL^−1^ IL‐4 and IL‐10, and 1 mg mL^−1^ dECM for 24 h, respectively. Then the cells were collected and stained using the following antibodies: APC anti‐mouse F4/80, FITC anti‐mouse, and PE anti‐mouse. Then fluorescence signals were detected by BD Accuri C6 (BD Biosciences).

### RNA Extraction and Real‐Time Polymerase Chain Reaction (RT‐PCR)

Total mRNA was extracted from cells with TRIzol reagent according to the manufacturer's instructions. RNA samples (500 ng) were then reverse transcribed to cDNA using PrimeScript RT reagent Kit (Takara, RR037A); reverse transcription was performed by 15 min incubation at 73 °C followed by 85 °C for 5 s and 4 °C for ∞ within the T100 thermal cycler (Bio‐Rad). RT‐PCR was performed by incubation at 95 °C for 30 s followed by 40 cycles of 95 °C for 10 s, 60 °C for 30 s on the 7500 Fast Real‐Time PCR System (Thermofisher) using ChamQ Universal SYBR qPCR Master Mix (Vazyme, Q711). Primer sequences used for mouse monocyte qRT‐PCR analysis are provided in Table S1 (Supporting Information).

### Extraction of Rat Cardiomyocytes and Fluorescence Staining of ROS

In a sterile environment, the hearts of ten neonatal SD rats (born within 24 h) were harvested, then immediately placed in sterile PBS and thoroughly washed to remove residual blood. The tissue was then cut into ≈1 mm^3^ pieces. A digestion enzyme mixture was prepared at a ratio of 0.08% trypsin and 0.06% type II collagenase (Gibco, 17101015). The tissue pieces were transferred into a 50 mL centrifuge tube, and 5 mL of the digestion enzyme mixture was added. After incubating at 37 °C in a water bath for 8 min, the supernatant was discarded. The remaining tissue was added to 10 mL of the digestion enzyme mixture and incubated at 37 °C with shaking for 10 min. The supernatant was collected by filtering into another 50 mL centrifuge tube, and digestion was terminated by adding an appropriate amount of FBS. The remaining tissue pieces were further digested 3–5 times until completely digested. All digestion solutions were collected in a centrifuge tube, and the mixture was centrifuged at 300 *g* for 10 minutes. The supernatant was discarded, and the cells were evenly plated in a six‐well plate. After incubating the six‐well plate in a 37 °C, 5% CO_2_ incubator for 90 min, the medium containing the cardiomyocytes was transferred to a new six‐well plate for further culture to obtain the cardiomyocytes. The adhered cardiomyocytes were incubated with H_2_O_2_ and different hydrogels for 24 h, followed by staining with DCFH‐DA probe.

### Rat Myocardial Infarction Model

Adult male Sprague Dawley rats (300–350 g) were anesthetized using 2%–3% isoflurane within an induction chamber. The incisional area was shaved using animal clippers and hair removal cream. Rats were intubated and ventilated with 0.75%–1.5% isoflurane and 100% medical‐grade oxygen (1 L min^−1^). The mechanical ventilation was set to a respiratory rate of 50 bpm, with a tidal volume calculated as the rat's weight (g) divided by 100, and an airway resistance ranging from 0 to 2 kPa. The chest skin was disinfected using a cotton ball saturated with 75% alcohol.

To induce MI, a thoracotomy was performed at the left fourth intercostal space, and the left anterior descending coronary artery (LAD) was permanently ligated with a 7‐0 polypropylene suture ≈2 mm below the left atrium's tip to create a large infarct area including the mid to apex portion of the left ventricular anterior wall. The infarction was verified through the blanching of the myocardial tissue and diminished movement in the ventricular wall region supplied by the LAD. After closing the chest and skin using 4‐0 silk sutures, the animal received oxygen support until fully awake and was placed under a heating lamp to aid in the recovery of body temperature. Sham animals followed the same procedure described above without LAD ligation and the suture placed beneath the LAD was subsequently removed.

To relieve pain and reduce the risk of infection, each animal received an intramuscular injection of 20000 U kg^−1^ penicillin and was administered 5 mg kg^−1^ of carprofen orally each day for three days following the surgery. The weight of the animals was closely monitored during this period.

### Electrocardiography

Electrocardiograms (ECGs) were simultaneously recorded with echocardiography using the Vinno D6 LAB at baseline before MI, pre‐treatment (day 0), and post‐treatment (day 7 and day 28). In addition, during the rat myocardial infarction modeling procedures, ECGs were captured using the Medlab‐U/4c501 biological signal acquisition and processing system. The procedures involved leads attached to all four limbs to evaluate the extent of myocardial infarction following coronary artery ligation.

### Injection

One week after MI, rats with a fractional shortening (FS) between 25% and 35% were randomly divided into four groups: BCM, ALG, dECM (*n* = 8 per group), and saline groups (*n* = 5).^[^ ^41^ ^]^ Seven days post‐MI, the heart function was accessed. A total of 100 µL of either hydrogel or saline was injected into the infarct zone at two randomly chosen sites (50 µL per injection) using two 1 mL syringes equipped with a 27G needle, all administered via a syringe pump. The needle was left in place within the myocardium for 5 s after each injection to minimize hydrogel leakage.

### Echocardiography

Cardiac function was evaluated using transthoracic echocardiography (Vinno D6 LAB) at baseline before MI, pre‐treatment (day 0), and post‐treatment (day 7 and day 28). Anesthesia was induced with 2%–3% isoflurane via an induction chamber and maintained with 0.75%–1.5% isoflurane with 1 L min^−1^ fresh gas flow via a mask. Most rats had heart rates between 380 and 420 bpm during the process of performing echocardiography. Animals were positioned supine, and the chest fur was removed using hair removal cream. Echocardiography was performed on the parasternal long‐axis view and left ventricle short‐axis view in B‐mode and M‐mode. Cardiac parameters assessed included EF, FS, LVAW, and LVPW, left ventricular anterior and posterior wall thickness ratio, left ventricular internal diameter at end‐diastole (LVIDd) and end‐systole (LVIDs). These evaluations were performed by an operator who was blinded to the group allocations of the animals. The value for each parameter was calculated by taking the average of measurements from three randomly selected cardiac cycles.

### Tissue Collection

On day 28 after the treatment, following echocardiography measurements, the animals were anesthetized with 5% isoflurane, and access to the heart was gained via thoracotomy. A small incision was made in the right atrium using ophthalmic scissors, and the heart was then perfused with 5 mL of 0.1 mol L^−1^ potassium chloride through the apex of the left ventricle using a 5 mL syringe. This procedure arrested the heart in diastole, and the ascending aorta was clamped using a vascular clamp to ensure the solution's distribution. Then the heart was washed in 4 °C PBS and fixed in a 4% paraformaldehyde solution for 24 h. After fixation, the heart tissue samples were dehydrated with gradient alcohol and xylene and then embedded in paraffin blocks and cut into 6 µm thickness sections. To further elucidate the mechanisms underlying the effects of ALG, BCM, and dECM hydrogels on myocardial infarction in rats, whole transcriptome RNA sequencing was conducted using the collection of cardiac tissue from the apical region of 7 d post‐treatment MI rats.

### Histological Analysis

The paraffin sections were stained with Masson trichrome and H&E, following the manufacturer's instructions. The infarcted area of heart was calculated based on Masson trichrome staining, with collagen deposition area in blue and myocardial areas in red. For immunofluorescence staining, the cardiac sections were washed three times with PBS, then incubated with 10% goat serum for 30 min. The primary antibodies of F‐actin (1: 500), CD68 (1:1000), CD86 (1:200), CD206 (1:500), and α‐SMA (1:200) were added at 4 °C for overnight. After that, all samples were incubated in secondary antibodies for 1 h at room temperature. Finally, the nucleus was stained with DAPI. The images were photographed under fluorescent microscopy (CX43, Olympus, Japan).

### ANGPTL4 Expression in H9C2 Cells

H9C2 cells were cultivate the in a 48‐well plate, and when the cell fusion degree reached 80%, 0.2% dECM, 2% ALG, 0.2% dECM + 100 × 10^−6^ m H_2_O_2_ and to 2% ALG + 100 × 10^−6^ m H_2_O_2_ were added into each well separately and incubated for 8 h. After the co‐culture period, the supernatants were collected to assess the concentration of ANGPTL4 using a rat ANGPTL4 ELISA Kit (CZKEWEI), following the manufacturer's guidelines.

### ANGPTL4 Adsorption by Hydrogel

0.5 mL ALG, ALG@dECM hydrogels and 1 mL 2 µg mL^−1^ ANGPTL4 were added in a 48‐well plate and shaken at 37 °C for 24 h. Then the supernatants were collected to assess the concentration of ANGPTL4 secreted by the H9C2 cells using a rat ANGPTL4 ELISA Kit (CZKEWEI), following the manufacturer's guidelines.

### Statistical Analysis

All data were reported as means ± standard error of the mean. Two groups were compared using two‐sided Student's *t*‐tests. Comparisons among three or more groups were performed using one‐way ANOVA followed by Tukey's multiple comparisons test. All statistical analyses were carried out using GraphPad Prism 8 software. The tests applied were two‐sided, and a *p*‐value of less than 0.05 was considered to indicate statistical significance.

## Conflict of Interest

The authors declare no conflict of interest.

## Supporting information



Supporting Information

## Data Availability

Research data are not shared.
