# Supplementary material for: Fish Swim Bladder‐Derived ECM Hydrogels Effectively Treat Myocardial Ischemic Injury through Immunomodulation and Angiogenesis
Source: Adv Sci (Weinh). 2025 Apr 9;12(23):2500036. doi: 10.1002/advs.202500036 (PMC12199318; doi:10.1002/advs.202500036)
Supplement: Supplementary file 1 — Supporting Information [file ADVS-12-2500036-s001.docx]

Fish swim bladder-derived ECM hydrogels effectively treat myocardial ischemic injury through immunomodulation and angiogenesis

Yulong Fu^#^, Canran Gao^#^, Hailing Zhang, Jing Liu, Boxuan Li, Wei Chen, Xiuping Chen, Xue Lin*, Ligang Fang*, Zhihong Wang*

Y. L. Fu,

Institute of Transplant Medicine, School of Medicine, Nankai University, Tianjin 300071, China; Institute of Biomedical Engineering, Chinese Academy of Medical Sciences and Peking Union Medical College, Tianjin 300192, China

C. Gao, W. Chen, X. Lin, L.G. Fang,

Peking Union Medical College Hospital, Chinese Academy of Medical Sciences and Peking Union Medical College (CAMS&PUMC), Beijing, 100005, China

E-mail: [fanglgpumch@sina.com](mailto:fanglgpumch@sina.com); [seeingsea@gmail.com](mailto:seeingsea@gmail.com)

X. P. Chen,

State Key Laboratory of Quality Research in Chinese Medicine, Institute of Chinese Medical Sciences, University of Macau, Macao, China

H. L. Zhang, J. Liu, B. X. Li,

Institute of Biomedical Engineering, Chinese Academy of Medical Sciences and Peking Union Medical College, Tianjin 300192, China

Z.H. Wang

Institute of Transplant Medicine, School of Medicine, Nankai University, Tianjin 300071, China. E-mail: nkwangzhihong@nankai.edu.cn

# These authors contribute equally.

**Supplementary Material**

Figure 1. Rheology of alginate hydrogels of different algnite concentration.


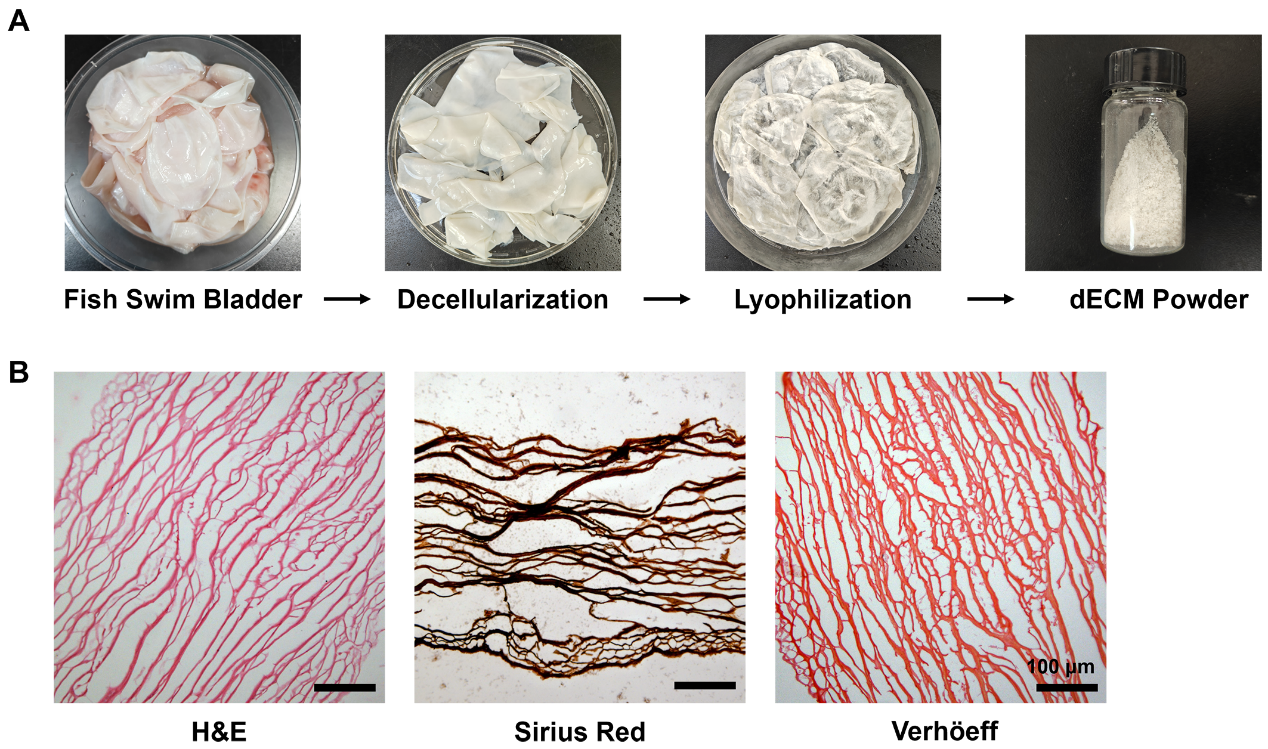


Figure 2. A, Preparation of decellularized fish swim bladder dECM. B, H&E, Sirius Red and Verhöeff staining of fish swim bladder post decellularization.


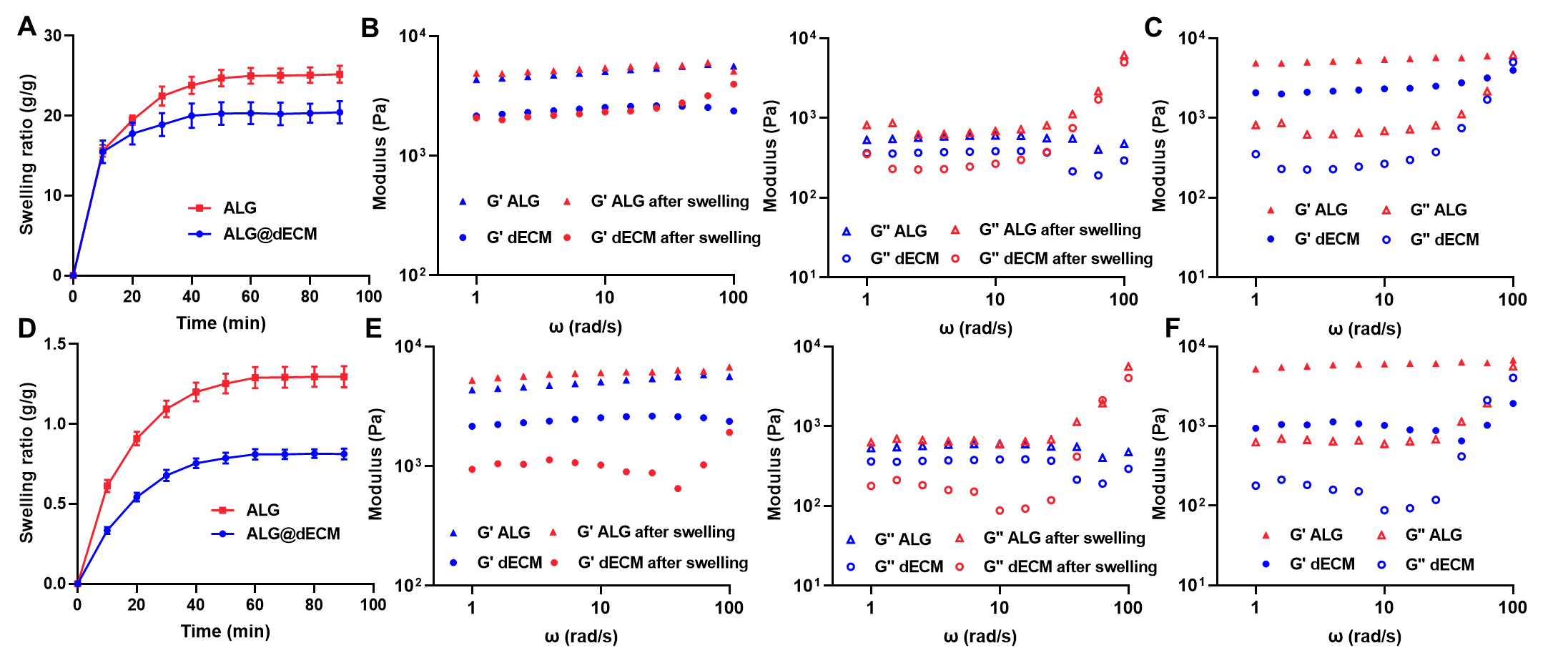


Figure 3. The swelling ratio and rheological property of ALG and ALG@dECM hydrogels. A, The swelling ratio in dry state. B, Rheology of ALG, and Alg@dECM hydrogels before and after swelling in dry state. C, Rheology of ALG, and Alg@dECM hydrogels and after swelling in dry state. D, The swelling ratio in hydrous state. E, Rheology of ALG, and Alg@dECM hydrogels before and after swelling in hydrous state. F, Rheology of ALG, and Alg@dECM hydrogels and after swelling in hydrous state. (mean ± SD, n = 5)


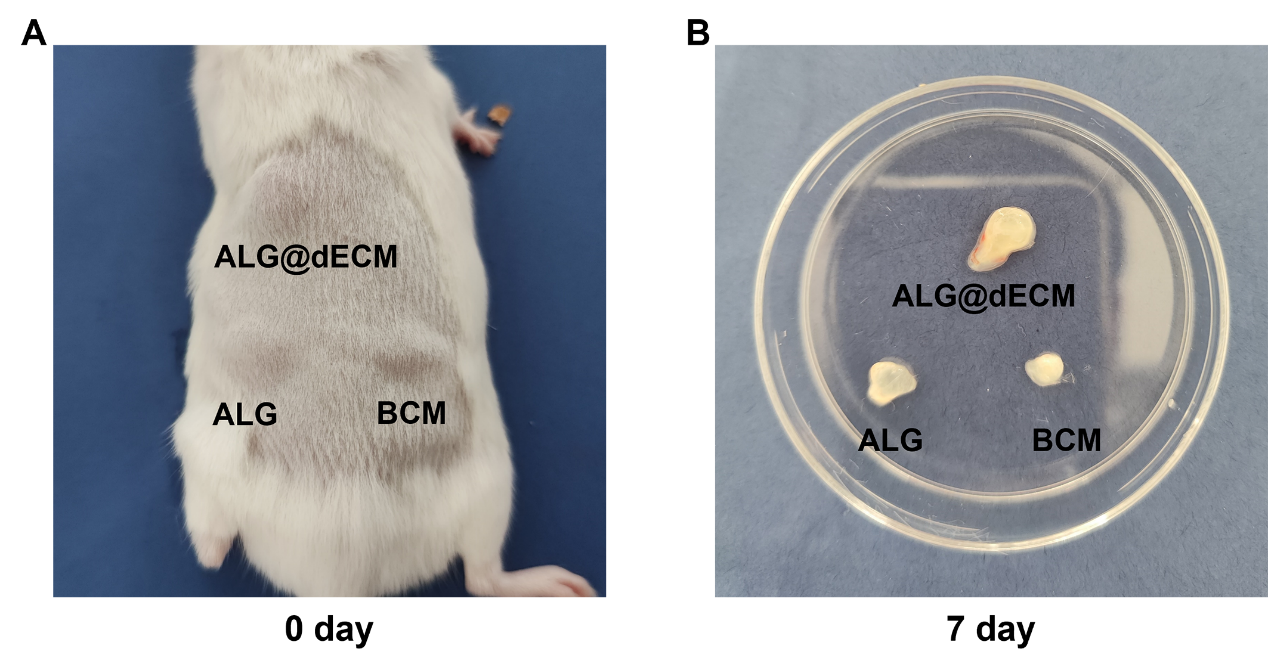


Figure 4. Pictures of hydrogel injected subcutaneously in mice.


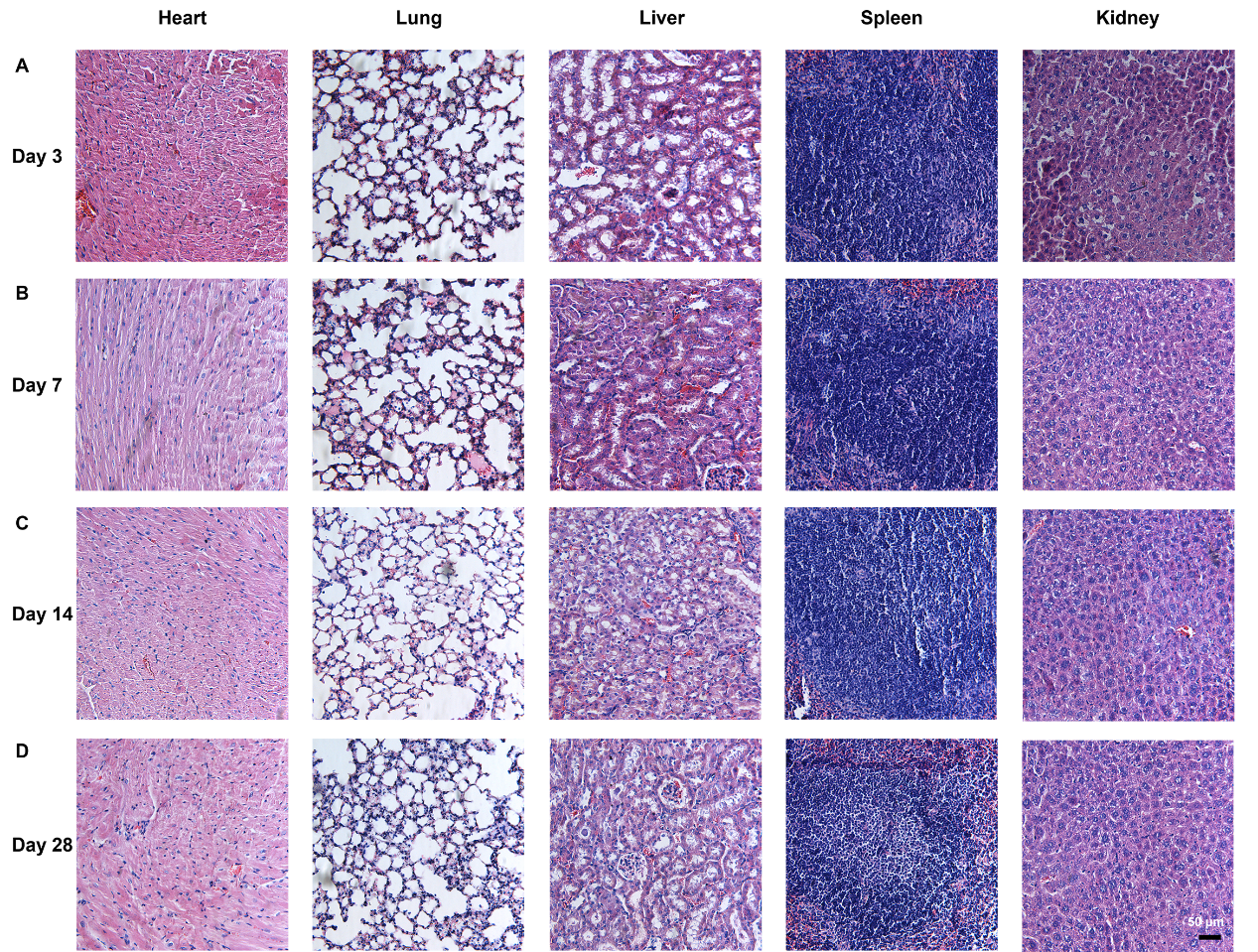


Figure 5. A-D, H&E staining of main organ of mice after the injection of ALG, BCM and ALG@dECM hydrogels in the subcutaneous implantation model at different time intervals.


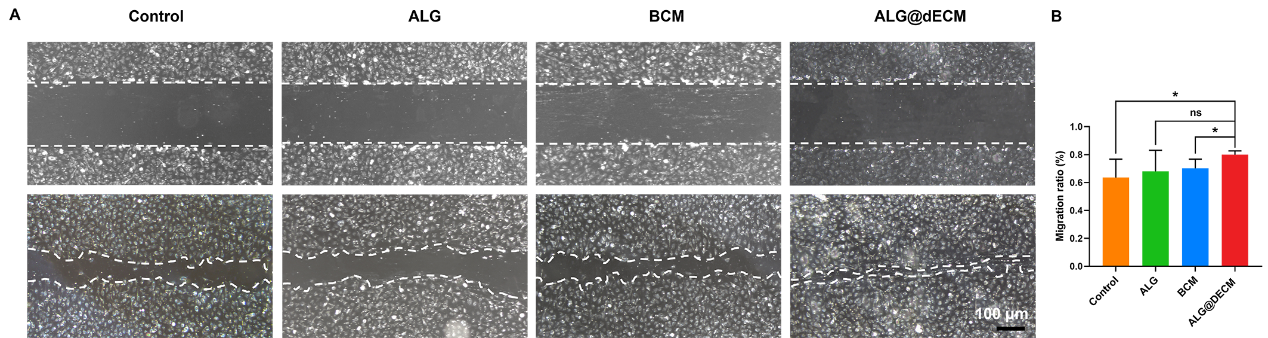


Figure 6. A-B，Wound healing of HUVECs cocultured with ALG, BCM and ALG@dECM hydrogel (n=5).


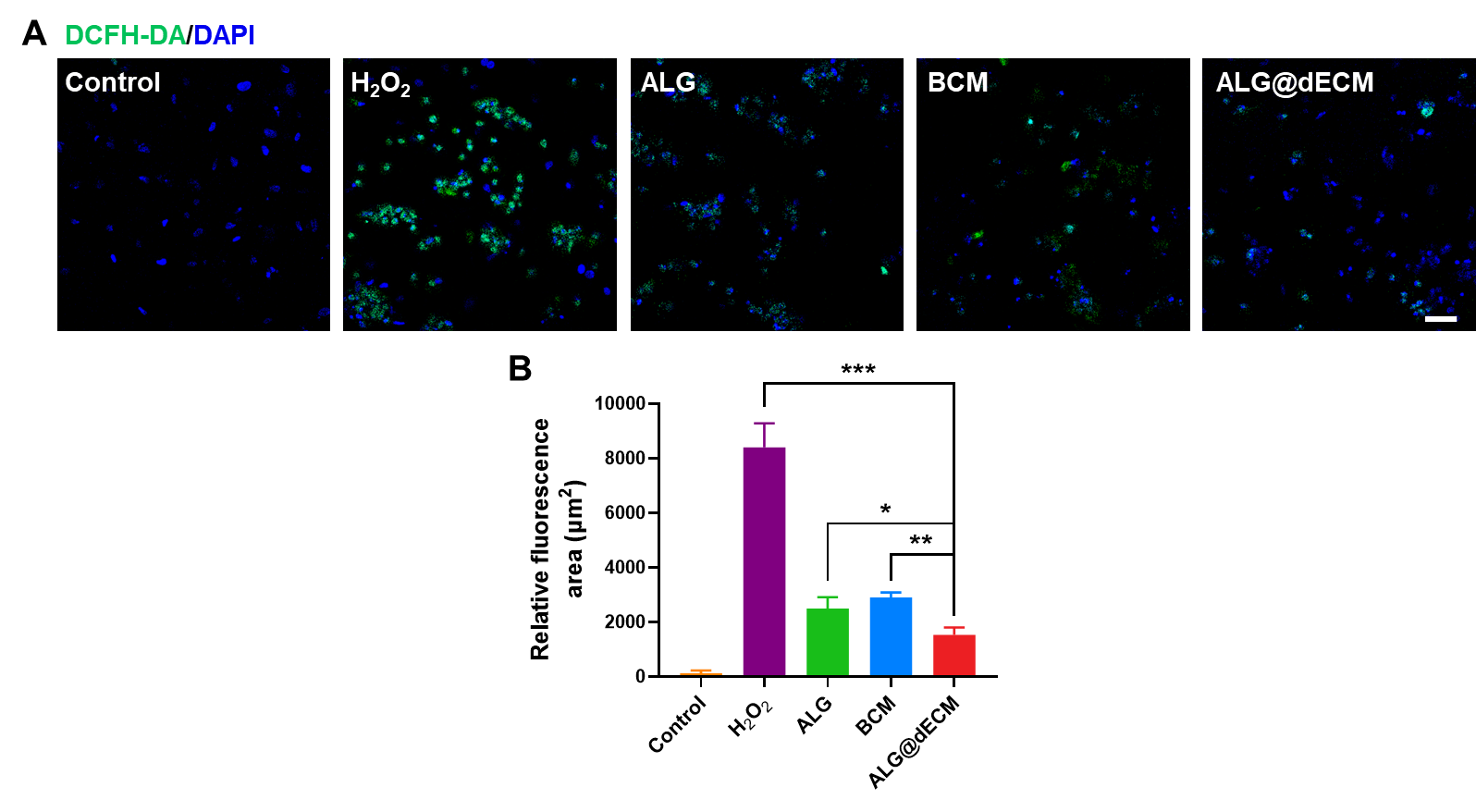


Figure 7. A, Representative fluorescent images of ROS (DCFH-DA) in rat primary cardiomyocytes. B, Quantification of DCFH-DA. (mean ± SD, n = 3, *p < 0.05 , **p < 0.01, and ***p < 0.001) Scale bar: 50 μm.

Figure 8. Quantification of wall thickness of left ventricular by Masson staining (Sham group, n=5; Saline group, n=5; ALG group, n=8; BCM group, n=8; ALG@dECM group, n=8).

Figure 9. Heart weight/ body weight of each group (Sham group, n=5; Saline group, n=5; ALG group, n=8; BCM group, n=8; ALG@dECM group, n=8).

| Gene | Forward primer sequence (5′–3′) | Reverse primer sequence (5′–3′) |
| --- | --- | --- |
| IL-6 | TAGTCCTTCCTACCCCAATTTCC | TTGGTCCTTAGCCACTCCTTC |
| TNF-α | CAGGCGGTGCCTATGTCTC | CGATCACCCCGAAGTTCAGTAG |
| IL-10 | GCTCTTACTGACTGGCATGAG | CGCAGCTCTAGGAGCATGTG |
| ARG-1 | CTCCAAGCCAAAGTCCTTAGAG | AGGAGCTGTCATTAGGGACATC |

Table S1. Primers used in qRT-PCR.
